# Direct and indirect predictors of medication adherence by adults with bipolar disorder

**DOI:** 10.1192/j.eurpsy.2022.1025

**Published:** 2022-09-01

**Authors:** B. Cohen, N. O’Rourke

**Affiliations:** 1 Ben-Gurion University of the Negev, Medicine, Be’er Sheva, Israel; 2 Ben-Gurion University of the Negev, Public Health, Be’er Sheva, Israel

**Keywords:** bipolar disorder, medication adherence, alcohol misuse, cognitive failures

## Abstract

**Introduction:**

Medication adherence by persons with bipolar disorder (BD) is inconsistent. This is disconcerting, as BD is treatment responsive, side-effects are few, and the impact of both hypo/manic and depressive mood episodes can be considerable (e.g., self-harm).

**Objectives:**

For this study, we computed a path model to identify both direct and indirect predictors of medication adherence. This included both clinical and psychosocial independent variables (e.g., BD symptoms, psychological well-being, alcohol misuse).

**Methods:**

From the BADAS (Bipolar Affective Disorder and older Adults) Study, we identified a global sample of adults with the BD. Participants were recruited using microtargeted, Facebook advertising. This sample included persons living in Canada, U.S., U.K., Ireland, Australia and New Zealand (M = 55.35 years, SD = 9.65).

**Results:**

Direct predictors included perceived cognitive failures and alcohol misuse. Of note, medication adherence is inversely associated with number of prescribed antipsychotic medications. Neither symptoms of depression nor hypo/mania emerged as direct predictors of medication adherence. Similarly, psychological well-being appears indirectly associated with adherence (via BD symptoms).

**Conclusions:**

Despite the wide age range of participants (22 – 73 years), age did not emerge as a predictor of adherence. Nor do cognitive failures appear significantly associated with age suggesting that both young and older adults with BD perceived cognitive loss.

**Disclosure:**

No significant relationships.

